# Protein supplementation preserves muscle mass in persons against sleeve gastrectomy

**DOI:** 10.3389/fnut.2024.1476258

**Published:** 2024-10-09

**Authors:** Nagehan Afsar, Yahya Ozdogan

**Affiliations:** ^1^Healthy Nutrition and Life Center, Ankara, Türkiye; ^2^Department of Nutrition and Dietetics, Faculty of Health Sciences, Ankara Yildirim Beyazit University, Ankara, Türkiye

**Keywords:** muscle mass, protein supplementation, sleeve gastrectomy, protein intake, postbariatric surgery diet

## Abstract

**Introduction:**

Sleeve gastrectomy surgery can lead to deficiencies in both macro and micronutrients, with protein being particularly crucial due to its role in muscle mass, physiological, and metabolic functions. Inadequate protein intake due to physiological, psychological, or financial reasons may prevent achieving the recommended intake levels. The significance of this issue is often underappreciated.

**Aim:**

This study evaluates the impact of protein supplementation on muscle mass in individuals undergoing sleeve gastrectomy and emphasizes the need for more comprehensive dietary training by expert dietitians.

**Method:**

Data were collected from 60 participants (15 male, 45 female, aged 20–54) who visited the surgery clinic. Participants were divided into two groups: those receiving the recommended protein supplement (15 g/day) with post-bariatric surgery diet training (BSD + PS), and those receiving only the post-bariatric surgery diet (BSD). A pre-surgery questionnaire gathered health and general information. Daily energy and nutrient intakes were recorded using 24-h food consumption logs on the day before surgery and at 7 days, 1 month, and 3 months postoperatively. Anthropometric measurements, including muscle and fat mass, and International Physical Activity Questionnaire (IPAQ) data were also collected.

**Findings:**

The characteristics of participants in both groups were similar, although there were more females in the BSD + PS group (86.7%) compared to the BSD group (63.3%). Despite an increase in energy and nutrient intake over time, levels remained below the recommended amounts in both groups. A significant difference was found in protein supplement consumption between the groups (*p* = 0.000). Repeated measures showed significant differences in body muscle mass percentage over time (*F* = 202.784; *p* = 0.000).

**Conclusion:**

In individuals who underwent sleeve gastrectomy surgery, deficiencies in macro and micronutrient intake were observed below reference levels. For this reason, the first approach in the treatment of obesity should always be medical nutrition therapy accompanied by a dietician. When designing post-bariatric surgery nutrition programs, it should be taken into consideration that nutrition protocols and trainings should be followed more closely and given in more detail under the supervision of a specialist before supplements are considered.

## 1 Introduction

Obesity is defined as a chronic and inflammatory disease that develops due to excess fat accumulation in the body, which can disrupt health and cause disease (1). The body mass index (BMI) used in the classification of obesity is calculated as the body weight in kilograms divided by the height in meters squared (2). According to BMI values, individuals over 25 kg/m^2^ are classified as having excess body weight, and individuals over 30 kg/m^2^ are classified as obese (1). Excess energy taken from food accumulates in many different parts of the body such as the liver and muscles and can cause diseases (3). The main ones of these diseases are metabolic diseases, heart diseases, psychosocial, central nervous system, reproductive system and pulmonary diseases (4). There are many different treatment options for obesity. These are grouped under 5 different headings: nutritional therapy, physical activity, lifestyle and behavioral changes, drug therapy and surgery (5). Treatment should always start with medical nutrition therapy, which is shown as the safest method. However, surgery should be considered in individuals who do not respond and meet the treatment criteria (6). The criteria that individuals who are considered suitable for surgical procedures will have are BMI > 40 kg/m^2^ or BMI 35–39.9 kg/m^2^ and obesity-related (prediabetes, diabetes, dyslipidemia, obstructive sleep apnea, asthma, polycystic ovary syndrome, infertility, sexual dysfunction, hypertension, atherosclerosis, impaired kidney function) having at least one concomitant disease or BMI 30–34.9 kg/m^2^ and concomitant diabetes and it is indicated in the form of metabolic syndrome (7).

The most basic purpose of surgical procedures is to reduce body weight and the health risks associated with obesity (8). There are 3 main procedures of bariatric surgery (BS): restrictive, absorbent and both restrictive and absorbent (9). Sleeve gastrectomy (SG), one of the surgical methods, is the vertical division of the stomach volume, leaving approximately one-fourth of it. In this process, the pyloric structure, gastric functions and digestive processes are not damaged (4). Reducing visceral fat in the body, accordingly, it is aimed to increase insulin sensitivity, reduce the amount of free fatty acids and interleukin-6 level (10). It has also been observed that their quality of life and psychosocial status have improved (11). However, there are also disadvantages. The most common complications are; leakage, hemorrhage, kidney stones, cholecystectomy, insufficient weight loss, liver or spleen injury, portal vein thrombosis, venous thromboembolism, breathing difficulties, abscess, stomach stenosis, venous thromboembolism, choledocholithiasis, pneumonia, sepsis, infection, minor complications and deaths. It can also cause macro and micronutrient deficiencies (12). It has also been observed that individuals regain weight after surgery due to the main reasons such as increasing the amount of energy consumed over time, choosing high-energy beverages, having five or more meals, insufficient activity level, advancing age, drug use or hormonal changes, increase in stomach volume, and high-carbohydrate diet (13).

When observational studies were examined, it was stated that the energy consumed should be similar to the general nutrition recommendations. It should be planned that 35–48% of the total energy should be met from carbohydrates, 37–42% from fat. Adequate and balanced nutrition recommendations should be determined by experienced dietitians during the day, the importance of high protein foods should be explained, and protein supplementation recommendations should be made if necessary (14). The most common macronutrient deficiency in all BS methods, especially in absorption-disrupting procedures, has been identified as protein (15). Protein deficiency is observed within the first month after surgical procedures, and the sensitivity to protein foods develops at the same rate (16). Protein deficiency is seen within the first month after surgical procedures, and sensitivity to protein foods develops at the same rate (16). This situation can occur in 3 main ways. The first is an aversion or feeling of nausea that develops against protein-rich foods due to changes in taste and smell. The second is the decrease in the amount of food taken due to the removal of a large part of the stomach volume. Finally, it is a decrease in the secretion of digestive enzymes, especially stomach acid and pepsinogen, and the passage of food through the digestive lumen very quickly (15, 17, 18). In addition, most patients after bariatric surgery are subjected to a solid liquid diet in the early period after surgery. They cannot consume a large amount of food at one meal or get solid protein in the first months. This indicates that there is a higher risk of developing protein malnutrition (19). It has been observed that inadequate protein intake can cause problems such as decreased skeletal muscle mass, low serum albumin level, endocrine imbalances, acceleration of the aging process, anemia, low immune system, bone and calcium losses, decrease in metabolic rate and failure to reach the target weight (20). A small amount of muscle mass loss can be expected in weight losses, but maintaining metabolic balance and muscle integrity is of vital importance (21).

Protein is an essential nitrogenous element necessary for all living life. It forms the building block of cells and enzymes that catalyze the metabolic reactions that take place (22). Protein is an essential nitrogenous element necessary for all living organisms. It is the building block of cells and enzymes that catalyze metabolic reactions (22). It is recommended as 0.8 g/kg/day for adults. The amount of intake can be changed during pregnancy and lactation, in growing children and in pathological conditions (23). Current guidelines recommend that patients consume 60–80 g protein per day or 1.2 g/kg of ideal body weight (24, 25). However, it has been reported that 45% of BS patients have problems in complying with these guidelines (26). A significant prevalence of lean body mass loss has been described in BS patients. Patients have been found to lose approximately 22% of their lean body mass within the first year after Laparoscopic Roux-en-Y gastric Bypass (RYGB) (27, 28). Moderately high protein diet intake has been found to preserve muscle mass and basal metabolic rate. However, it has also been found to support weight loss (29, 30). Considering these considerations, bariatric guidelines recommend the adoption of a relatively high protein intake in the first months after surgery, when the risk of energy restriction and associated loss of lean mass is higher (31, 32). During this period, the use of high-quality protein sources with high leucine content is of vital importance for many tissues and organs, especially muscle mass (33). When the literature was reviewed, it was observed that the number of studies on this subject was insufficient. The aim of the study was to evaluate the effect of protein supplementation recommended in addition to medical nutrition therapy on the muscle mass of patients after BS. To emphasize the importance of more comprehensive nutrition education before supplementation is considered.

## 2 Materials and methods

### 2.1 Selection criteria of individuals

It has been observed in general nutrition education after surgery that protein intake of individuals is inadequate due to reasons such as changed gastric capacity after surgery, development of food intolerances, difficulty in consuming supplements, difficulty in meeting protein requirements, unwillingness to continue supplementation routine and even refusal to use additional supplements. This study is a prospective study with follow-up aiming to evaluate the effect of protein supplementation in addition to post-bariatric surgery diet on muscle mass measurements in individuals undergoing sleeve gastrectomy (SG) surgery. The primary endpoint is to evaluate the effect of protein supplementation on the existing lean body mass due to protein deficiency in patients who underwent SG surgery. The primary outcomes were weight loss and body composition at days 7, 30 and 90 after SG. Variables assessed included weight, BMI, muscle mass, fat mass, fat percentage, percentage of muscle mass loss, and percentage of fat loss. Secondary outcomes were changes in macro- and micronutrient status at days 7, 30 and 90 after SG. As a result of the power analysis using the G*Power 3.0.10 program, the sample size was determined as at least 59 people in total with 80% power, 5% type 1 margin of error and *d* = 0.7500000 effect size. The study was conducted by collecting data from a total of 60 individuals, aged between 20–54 years, who received 30 post-bariatric surgical diet (BSD) and 30 post-bariatric surgical diet + protein supplements (BSD + PS) on the day before surgery (pre-op 0) and 7, 30 and 90 days after surgery (post op). The patients who have been decided to undergo SG surgery have been divided into two groups, consisting only of BSD and BSD + PS, due to their inability to comply with the use of protein supplements. Participants received verbal and written diet instructions during their hospital stay, as well as at 7, 30, and 90 days following their bariatric surgery. They were advised to strictly adhere to the post-bariatric diet guidelines provided by the ASMBS Allied Health Nutrition Committee. This ensured that both groups consumed a similar, safe diet throughout the study period. The study was approved by the Ethics Committee of Ankara Yildirim Beyazit University with the decision number 40 dated 08.12.2020, permission was obtained from the clinic where the study was conducted, and a voluntary consent form was obtained from the participating patients. The study was conducted in Ankara General Surgery Clinic between January and June 2021.

All individuals scheduled for surgery were evaluated multidisciplinary by physicians, dietitians, and anesthesiologists according to their comorbidities. All procedures were performed by a single physician in accordance with the relevant guidelines and regulations. Individuals with body mass index BMI ≥ 40 kg/m^2^ or BMI ≥ 35 kg/m^2^ and at least one comorbidity associated with obesity (prediabetes, diabetes, dyslipidemia, obstructive sleep apnea, asthma, polycystic ovary syndrome, infertility, sexual dysfunction, hypertension, atherosclerosis, impaired renal function) were included in the study. Individuals who had not achieved body weight loss for at least 6 months on various diet programs and had the capacity to understand the surgical procedure and its effects were selected. The exclusion criteria for this study are as follows;

- Under 19 or over 64 years old
- Having uncontrolled endocrinological diseases
- Pregnancy
- Having uncontrolled mental health problems or depression
- Having substance addiction
- Having problems that cannot follow the guidelines and recommendations regarding nutritional status
- Having eating disorders
- Having cancer
- Having coagulation disorders
- Having contraindications that prevent surgery (cardiovascular disease, anesthesia-related risks, etc.)

Demographic characteristics, general and health information (gender, age, education level, family history of obesity, consumption of beverages with added sugar, tea consumption, use of added sugar, sleep duration, alcohol consumption, chronic disease status, methods used to reduce body weight, night eating habits, water consumption) were questioned with a questionnaire.

When the studies conducted on this subject are examined, it is seen that a follow-up period of up to 3, 6, 12, or 36 months is planned (32). However, due to the pandemic experienced worldwide, the study was continued for 3 months in order to complete the process (34).

### 2.2 Protein supplement

It is known that individuals develop many macro and micronutrient deficiencies due to stomach capacity and possible physiological changes, and protein is defined as the most important nutrient (16). Considering the importance of protein intake, recommendations are made by the American Association of Clinical Endocrinologists, the Obesity Society, and the American Society for Metabolic and Bariatric Surgery (ASMBS). It is emphasized that these recommendations should be individualized, evaluated, and recommendations should be made by a dietitian who is an expert in the field, recording the gender, age, and current body weight of the individuals. Protein intake should be a minimum of 60 g per day (35).

Individuals who complete the surgical process stay in the hospital for 2 nights and 3 days. In the clinic routine, post-bariatric surgery diet education is started to be given (by the dietitian) in the morning visits. These medical nutrition programs are created specifically for individuals in accordance with the literature and necessary recommendations are made (36). 24-h food consumption records were evaluated using a food photo catalog in the pre-op 0 and post-op control periods. These data were analyzed using the “Computer-Assisted Nutrition Program, Nutrition Information System” (BeBis) (37). The nutrient values calculated via BeBis were classified according to the ‘Dietary Reference Intake Level’ (DRI) recommendations according to age and gender % ≤67 of the reference values were categorized as inadequate, % 67–133 as sufficient and ≥ %133 as excessive intake (38). In addition to the nutrition programs, the recommended protein supplement was planned to be consumed without cooking by adding it to foods in liquid form with the help of a scale once a day. Those who consumed less than 80% of the supplement were excluded from the study. A similar study was taken as an example and 15 g/day whey protein supplement was added (39). Because individuals who have undergone bariatric surgery may develop an aversion to protein-rich foods due to changes in taste and smell. In addition, removal of a large portion of the stomach volume may reduce the amount of food taken in and the release of enzymes that help digestion and may cause physiological changes that reduce the amount of food intake (17).

#### 2.2.1 Educational content of post-bariatric surgery diet

The content of the nutrition education provided to postoperative patients is as follows (40):

##### 2.2.1.1 Post-op 1–2, day (hospital admission process)

- To begin with, clear liquids (sugar, carbohydrate and caffeine-free) should be consumed.
- It should be switched to liquid intake as sipping and as tolerance is achieved, it should be ensured to consume liquid in a way that can be approximately 1,400 mL per day.
- The use of straws should always be avoided to prevent the formation of air bubbles.

##### 2.2.1.2 Post-op 3–7, day (discharged)

- The consumption of clear liquids (sugar, carbohydrate and caffeine-free) should be maintained.
- However, approximately half of the recommended daily consumption of 1,400 mL-1800 mL of liquid should consist of clear liquid.
- It should be switched to full liquids (skimmed milk, lactose-free milk, soy milk, plain yogurt or grain-free soup).
- However, whey or soy-based protein powder can be added to complete liquids (<20 g/meal).
- A chewable multivitamin and mineral supplement should be started, such as a tablet twice a day.

##### 2.2.1.3 Post-op 2–3, week (puree diet)

- Clear liquids can be increased up to a daily amount of 1,400–1800 mL.
- Soft, pureed, ground solid foods with a low fat and high protein content should be added instead of full liquids (eggs, cheese with a low-fat content, fish, poultry, lean meat, boiled beans).
- It should be consumed with 4 or 6 meals during the day, and the meal portion should be approximately <60 mL/meal planned.
- First of all, protein should be consumed. Daily protein consumption of 60 g and above should be targeted.

##### 2.2.1.4 Post-op 4–6, week

- As long as tolerance is achieved, the nutritional steps should progress. Well-cooked vegetables, soft or crushed, peeled fruits can be included in nutrition programs.
- 4–6 meals can be consumed daily, and the meal portions should be approximately 120 mL/meal.
- First of all, meals should be started by consuming protein foods.
- To avoid the risks of dehydration, 1,400–1,800 mL of clear liquid should be consumed daily.
- Drinks should not be consumed for 30 min before meals and for 30–60 min after meals.
- Besides, food should be chewed well.

##### 2.2.1.5 Post-op 7, the week and beyond

- Height length, body weight and age factors should be planned by evaluating daily energy requirements.
- A balanced nutrition program should be established in which lean meat products, fruits, vegetables and whole grains are added.
- The consumption of vegetables and fruits with high fiber density should be avoided. These products can be consumed well-cooked or mashed.
- A daily nutrition plan should be created so that three main meals and two intermediate meals are consumed. The serving size should not be over 240 mL.
- The daily consumption of clear liquid in the amount of 1,400–1,800 mL should be ensured.
- A Drink should not be consumed 30 min before meals and should be planned to be consumed 30–60 min after.
- On the other hand, it is planned that foods should be chewed well (26).
- Any diet application before surgery is not recommended, but nutrition education based on literature is provided. After the surgery, face-to-face nutrition education is repeated practically during the visiting hours of the patients.

#### 2.2.2 General nutrition education

Important points to be considered in the nutrition of postoperative patients are explained below (36).

- It is recommended to reach a protein amount of 60–120 g/day in order to maintain body muscle mass. This value is for RYGB; 1.1–1.5 g/kg/day and for BPD; care should be taken to take the amount of 120 g/day,
- To avoid simple sugar (sucrose) products and foods with low nutrient content and high energy content in order to prevent dumping syndrome that may occur after absorbent or restrictive procedures, avoid simple sugar (sucrose) products and foods with high energy content,
- Avoiding caffeinated and acidic drinks, fried foods, alcohol, foods containing high saturated fat,
- They should consume 3–6 meals daily, take small bites, chew well before swallowing, and meals should last about 30 min,
- It is in the form of taking multivitamin supplements in chewable or liquid forms for at least 6 months after surgery.

### 2.3 Anthropometric measurement analysis

Body composition was evaluated with bioelectrical impedance (TANİTA 780 MA) analysis under the supervision of a dietician. The individuals who will be measured were informed about the points they should pay attention to before coming for measurement. The main points are; not to consume food and beverages including coffee and tea with diuretic effects for 2 h before the measurement, not to do intense physical activity, not to consume alcohol, not to have any metal objects in contact with their skin during the measurement (41). Anthropometric measurements, mainly body weight, muscle and fat mass, were repeated in appropriate forms at the end of the pre-op and post-op 7 days, at the end of the 1st and 3rd months.

### 2.4 Assessment of physical activity

The International Physical Activity Questionnaire (IPAQ-Short Form), which was developed by the International Physical Activity Assessment Group and whose Turkish validity and reliability was performed by Öztürk (42) in 2005, was used to determine the physical activity status of individuals. IPAQ includes subheadings that determine the MET score by evaluating the frequency, duration and physical intensity levels of physical activity performed in the last seven days. The short form is calculated by multiplying the duration minutes and frequency data of walking, moderate activity and vigorous activity.

### 2.5 Statistical evaluation

In order to compare quantitative variables, it was first investigated whether parametric test conditions were met. In the comparison of quantitative data with normally distributed data, independent *t* test analysis was applied for the difference between two independent groups. Mann Whitney U test was applied for the difference between two independent groups that did not show normal distribution. Chi-square analysis was used to measure the relationship between categorical variables. In our study, qualitative variables were summarized by number and percentage, and measurements related to quantitative variables were summarized by mean and standard deviation. The obtained measurements were analyzed by repeated measures analysis of variance (ANOVA). These models were tested for main effects (group and time) and interaction effect (group*time). In such models, a significant interaction effect is interpreted as evidence that time-dependent changes differ across groups and main effects are not interpreted. In cases where the interaction effect is not significant, the results regarding the main effects are interpreted. In all analyses, the significance level was accepted as 5% and all analyses and graphs were performed using “IBM SPSS v25 for Windows (IBM Corp, Armonk, NY, USA)” software.

## 3 Results

### 3.1 Sociodemographic conditions

This study was conducted with a total of 60 individuals who received protein supplementation in addition to post-bariatric surgery diet (BSD + PS) and post-bariatric surgery diet alone (BSD) after bariatric surgery. In the BSD + PS group, 26 (86.7%) were female and 4 (13.3%) were male, while in the BSD group, 19 (63.3%) were female and 11 (36.7%) were male. In both groups, it was observed that the majority of those who wanted to have surgery were women. The mean age of those in BSD + PS was 33.6 ± 10.4 years, while the mean age of those in BSD was 36.3 ± 9.9 years. When the education levels were analyzed, 16 (53.3%) of the individuals in BSD + PS were undergraduates and 16 (53.3%) of those in BSD were high school graduates. The educational level of individuals in BSD + PS was found to be higher (*p* < 0.05). The majority of those in BSD + PS and BSD had a family history of obesity. Eighteen (69.2%) of those in BSD + PS and 17 (75.0%) of those in BSD were obese. The distribution of the patients included in the study is shown in Table 1.

**Table 1:** Distribution of sociodemographic, health, and nutritional status of individuals.

Gender sex, *n* (%)	BSD + PS *n* (%)	BSD *n* (%)	*p*
Woman	26.0 (86.7)	19.0 (63.3)	0.037*
Male	4.0 (13.3)	11.0 (36.7)	
Age (year)			
Avrg. ± Std. deviation	33.6 ± 10.44	36.3 ± 9.95	
Education level *n* (%)			
Middle School	3.0 (10)	7.0 (23.3)	0.025*
High School	9.0 (30.0)	16.0 (53.3)	
License	16.0 (53.3)	5.0 (16.7)	
Postgraduate	2.0 (6.7)	2.0 (6.7)	
Family history of obesity *n* (%)			
There is	26.0 (86.7)	24.0 (80.0)	0.488
No	4.0 (13.3)	6.0 (20.0)	
Sugary drink consumption (330 mL) cans/day			
Yes	22.0 (73.3)	19.0 (63.3)	0.405
No	8.0 (26.7)	11.0 (36.7)	
Tea consumption (120 mL)/ medium-sized tea cup			
Yes	21.0 (70.0)	27.0 (90.0)	0.053*
No	9.0 (30.0)	3.0 (10.0)	
Frequency of added sugar consumption (qty/cube/day)			
Does not consume	16.0 (53.3)	17.0 (56.7)	0.462
1–5 pieces	6.0 (20.0)	9.0 (30.0)	
6–10 pieces	4.0 (13.3)	1.0 (3.3)	
11 Pieces and above	4.0 (13.3)	3.0 (10)	
Sleep Duration (hours)			
Mean ± Std. deviation	7.3 ± 1.80	7.8 ± 2.19	0.482
Alcohol consumption			
Yes	10.0 (33.3)	7.0 (23.3)	0.390
No	20.0 (66.7)	23.0 (76.7)	
Chronic disease status			
Yes	25.0 (83.3)	20.0 (66.7)	0.136
No	5.0 (16.7)	10.0 (33.3)	
Previous methods to reduce body weight**			
Diet	30.0 (100.0)	30.0 (100.0)	0.076
Exercise	25.0 (83.3)	23.0 (76.7)	0.519
Acupuncture	8.0 (26.7)	10.0 (33.3)	0.573
Gastric balloon	-	1.0 (3.3)	0.313
Stomach Botox	1.0 (3.3)	1.0 (3.3)	0.754
Diet implementation			
Nothing	-	-	0.238
1–10	21.0 (70.0)	21.0 (70.0)	
11–20	7.0 (23.3)	5.0 (16.7)	
21 and above	2.0 (6.7)	4.0 (13.3)	
Method of providing dietary support**			
Dietitian support	22.0 (73.3)	24.0 (80.0)	0.542
Internet book recommendations	15.0 (50.0)	11.0 (36.7)	0.297
Individual effort	20.0 (66.7)	20.0 (66.7)	0.608
Medication or supplements	8.0 (26.7)	14.0 (46.7)	0.108
Night eating habits			
Yes	21.0 (70.0)	18.0 (60.0)	0.417
No	9.0 (30.0)	12.0 (40.0)	
Types of food consumed at night**			
Cheese and derivatives	2.0 (9.5)	1.0 (5.6)	0.643
Meat products	1.0 (4.8)	9.0 (50)	0.348
Bakery products	9.0 (42.9)	-	0.656
Nuts	3.0 (14.3)	1.0 (5.6)	0.370
Drinks with added sugar	1.0 (4.8)	2.0 (11.1)	0.458
Processed convenience foods	5.0 (23.8)	8.0 (44.4)	0.173
Water consumption (200 mL) cups/day			
Mean ± SD deviation	1146.6 ± 1164.04	1253.3 ± 1147.93	0.442

Chi-square analysis. *p values considered statistically significant are indicated in bold (*p* < 0.05), Post-bariatric Surgery Diet Group (BSD), Post-bariatric Surgery Diet Group + Protein Supplement Group (BSD + PS) **more than one option is marked.

### 3.2 Nutrition habits

According to the answers given to the question about eating speed, it was found that fast eating habits were common in 26 (86.7%) and 27 (90%) individuals in BSD + PS and BSD. When sugary drink consumption was analyzed, 22 (73.3%) of the individuals in BSD + PS and 19 (63.3%) of those in BSD answered yes to the question. The majority of individuals in both groups consume tea. The difference between the frequency of tea consumption and BSD + PS and BSD groups was statistically significant. When the consumption of added sugar was questioned, it was found that 6 (20.0%) individuals in BSD + PS and 9 (30.0%) individuals in BSD consumed 1–5 pieces of added sugar daily. However, the majority of individuals in BSD + PS and BSD did not use added sugar.

### 3.3 Medical history conditions

The mean sleep duration of the individuals in BSD + PS was 7.3 ± 1.8 h, while the mean sleep duration of those in BSD was 7.8 ± 2.1 h. The majority of the individuals in both groups did not smoke. When alcohol consumption was analyzed, it was found that 10 (33.3%) of the individuals in BSD + PS and 7 (23.3%) of those in BSD consumed alcohol. The majority of the individuals belonging to both groups, 25 (83.3%) of the individuals in BSD + PS and 20 (66.7%) of those in BSD, had at least one chronic disease. Individuals belonging to both groups made attempts to reduce their body weight before surgery. However, these interventions differed between the groups. Among the individuals in BSD + PS, 30 (100.0%) diet, 25 (83.3%) exercise, 8 (26.7%) acupuncture and among those in BSD, 30 (100.0%) diet, 23 (76.7%) exercise and 10 (33.3%) acupuncture were used to reduce or control their weight. The majority of the individuals belonging to both groups stated that they applied diet 1–10 times (Table 1).

### 3.4 Evaluation of anthropometric measurements

It was observed that the mean percentages of muscle mass before surgery decreased at the end of the 7th day (50.4, 53.8%) in BSD + PS (51.7%) and BSD (54.5%), respectively, and the mean percentages of fat mass increased in BSD + PS (45.6%) and BSD (42.7%), respectively, compared to the beginning (46.9, 43.4%). These percentage changes indicate the changing percentages in the current weight due to sudden muscle loss in the first seven days. However, the mean percentage of fat mass decreased, and the mean percentage of muscle mass increased in the current weight of the men and women belonging to the groups over time (Table 2). Individuals belonging to both groups had a high mean percentage of muscle mass within their current body weight at baseline. There was a statistically significant difference between BSD and BSD + PS at pre-op and post-op 7th, 30th and 90th days (*p* < 0.05). The mean percentage of available muscle mass of individuals in BSD + PS (58.2%) was lower than those in BSD (61.4%). When women and men were evaluated, the percentage of fat mass in the current weight of women in both groups was found to be higher in all periods compared to men (Table 2).

**Table 2:** Mean and standard deviation (X̄ ± SS) values of anthropometric measurements for general and gender.

		BSD + PS (*n* = 30)	BSD (*n* = 30)	*p*	BSD + PS (*n* = 30)	BSD (*n* = 30)	*p*	BSD + PS (*n* = 30)	BSD (*n* = 30)	*p*
		X̄ ± SS	X̄ ± SS		X̄ ± SS	X̄ ± SS		X̄ ± SS	X̄ ± SS	
		General			Female			Male		
Body weight (kg)	Pre-Op	111.6 ± 19.77	119.4 ± 24.01	0.178	106.9 ± 16.11	111.5 ± 18.21	0.375	142.7 ± 11.25	133.1 ± 27.38	0.512
	Post Op 7. Days	104.8 ± 18.87	112.3 ± 22.82	0.170	100.3 ± 15.47	105.8 ± 17.94	0.286	134.2 ± 10.95	123.6 ± 26.65	0.462
	Post Op 30. Days	98.9 ± 16.98	105.2 ± 19.51	0.189	94.8 ± 13.41	99.3 ± 17.61	0.334	125.3 ± 14.67	115.3 ± 19.22	0.362
	Post Op 90.Days	86.4 ± 16.09	92.2 ± 17.81	0.187	82.3 ± 12.73	87.7 ± 15.56	0.206	112.7 ± 9.25	100.0 ± 19.49	0.239
BMI (kg/m^2^)	Pre-Op	41.1 ± 4.97	42.8 ± 6.35	0.245	40.4 ± 4.95	42.3 ± 5.65	0.252	45.1 ± 3.18	43.7 ± 7.61	0.733
	Post Op 7. Days	38.5 ± 4.84	40.3 ± 6.10	0.218	37.9 ± 4.83	40.2 ± 5.55	0.157	42.4 ± 3.16	40.5 ± 7.24	0.635
	Post Op 30. Days	36.3 ± 4.28	37.8 ± 5.43	0.260	35.9 ± 4.34	37.8 ± 5.56	0.224	39.4 ± 2.29	37.9 ± 5.22	0.589
	Post Op 90. Days	31.7 ± 4.09	33.1 ± 5.08	0.234	31.1 ± 3.93	33.3 ± 4.98	0.105	35.6 ± 3.05	32.8 ± 5.48	0.365
Current fat mass (kg)	Pre-Op	50.9 ± 10.66	51.0 ± 13.83	0.982	50.1 ± 11.01	53.3 ± 12.68	0.380	56.1 ± 6.86	47.1 ± 15.43	0.287
	Post Op 7. Days	49.2 ± 10.22	49.0 ± 14.44	0.963	48.6 ± 10.61	51.8 ± 13.13	0.367	53.0 ± 7.04	44.2 ± 15.94	0.313
	Post Op 30. Days	43.3 ± 9.59	43.2 ± 13.10	0.968	43.2 ± 9.61	46.1 ± 12.85	0.390	44.2 ± 10.8	38.2 ± 12.52	0.414
	Post Op 90. Days	33.7 ± 8.84	33.1 ± 12.28	0.846	33.4 ± 8.88	37.0 ± 11.48	0.240	35.6 ± 9.58	26.5 ± 11.1	0.170
Percent fat mass (%)	Pre-Op	45.6 ± 4.34	42.7 ± 7.44	0.073	46.5 ± 3.63	47.3 ± 4.22	0.520	39.3 ± 3.50	34.7 ± 4.34	0.080
	Post Op 7. Days	46.9 ± 4.53	43.4 ± 8.07	0.043*	48.0 ± 3.42	48.3 ± 4.55	0.823	39.5 ± 4.10	34.9 ± 5.14	0.133
	Post Op 30. Days	43.8 ± 5.36	40.8 ± 8.29	0.105	45.1 ± 3.95	45.6 ± 5.19	0.714	35.0 ± 5.28	32.4 ± 5.47	0.442
	Post Op 90. Days	38.7 ± 5.87	35.2 ± 10.16	0.103	39.9 ± 5.02	40.8 ± 7.34	0.644	31.4 ± 6.28	25.5 ± 6.31	0.133
Current muscle mass (kg)	Pre-Op	57.7 ± 11.49	65.0 ± 15.71	0.043*	53.8 ± 5.56	55.2 ± 6.07	0.437	82.6 ± 8.13	82.0 ± 12.28	0.930
	Post Op 7. Days	50.1 ± 10.31	60.2 ± 14.29	0.031*	49.1 ± 5.30	51.3 ± 5.31	0.184	77.4 ± 8.44	75.7 ± 11.21	0.787
	Post Op 30. Days	52.8 ± 10.87	59.0 ± 30.286	0.049*	49.0 ± 4.36	50.5 ± 5.07	0.284	77.4 ± 7.38	73.5 ± 7.86	0.412
	Post Op 90. Days	52.9 ± 11.29	56.2 ± 20.059	0.043*	46.5 ± 4.26	48.2 ± 4.87	0.228	73.5 ± 5.77	70.2 ± 8.71	0.496
Percent muscle mass (%)	Pre-Op	51.7 ± 4.17	54.5 ± 7.19	0.071	50.8 ± 3.44	50.0 ± 3.98	0.511	57.8 ± 3.33	62.2 ± 4.16	0.081
	Post Op 7. Days	50.4 ± 3.95	53.8 ± 6.97	0.313	49.3 ± 3.27	49.1 ± 4.33	0.314	57.7 ± 3.86	62.1 ± 5.96	0.664
	Post Op 30. Days	53.4 ± 5.13	56.3 ± 8.01	0.100	52.1 ± 3.73	51.6 ± 4.91	0.713	62.0 ± 5.97	64.5 ± 5.27	0.065
	Post Op 90. Days	58.2 ± 5.76	61.4 ± 9.61	0.124	57.1 ± 4.96	55.8 ± 6.11	0.850	65.4 ± 6.00	71.1 ± 6.01	0.309

The “Independent Sample-*t*” test (*t*-Chart value) statistics were used to compare two independent groups with normal distribution. *The *p* values considered statistically significant are indicated in bold (*p* < 0.05). BMI, Body Mass Index; BSD, Post-bariatric Surgery Diet Group; BSD + PS, Post-bariatric Surgery Diet Group + Protein Supplement Group.

### 3.5 Evaluation of lost anthropometric measurements

**Figure 1:**
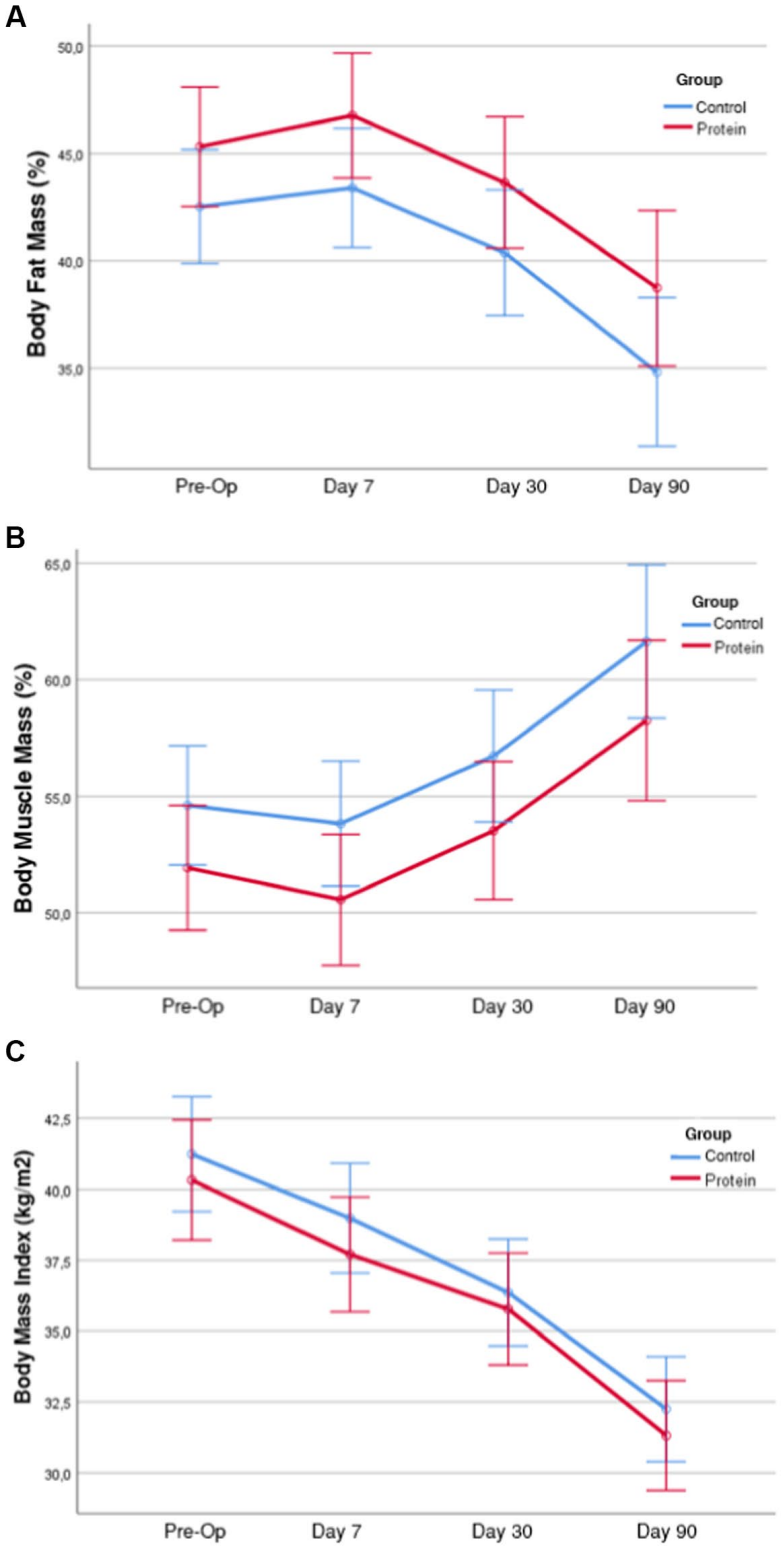
Time dependent changes and comparisons of body fat mass (%), muscle mass percentage, body mass index in groups, body fat mass (%) measurement averages of the groups by time (with 95% Interval). **(A)** According to the results of the analysis of variance of the body fat mass (%) variable, the group-time interaction effect was not significant (*F* = 0.692; *p* = 0.558). Therefore, the time-dependent changes in body fat mass (%) were similar in the groups. When the main effects were analyzed, the difference between the groups was not significant (*F* = 2.586; *p* = 0.115) and the time-dependent changes were significant (*F* = 173.34; *p* < 0.001). In short, while the time-dependent changes in Body Fat Mass (%) were significant in both groups, the difference between the groups was not significant. **(B)** According to the results of the analysis of variance of the body muscle mass (%) variable, the group-time interaction effect was not significant (*F* = 0.339; *p* = 0.754). Therefore, the time-dependent changes in body muscle mass (%) were similar in the groups. When the main effects were analyzed, the difference between the groups was not significant (*F* = 2.452; *p* = 0.125) and the time-dependent changes were significant (*F* = 192.10; *p* < 0.001). In short, in the variable body muscle mass (%) of the groups, the time-dependent changes were significant in both groups, but the difference between the groups was not significant. **(C)** According to the variance analysis results of the BMI variable, the group-time interaction effect was not significant (*F* = 0.917; *p* = 0.435). Therefore, the time-dependent changes of BMI variables in the groups were similar. When the main effects were analyzed, the difference between the groups was not significant (*F* = 0.468; *p* = 0.498) and the time-dependent changes were significant (*F* = 667.85; *p* < 0.001). In short, while time-dependent changes were significant in both groups in the BMI variable of the groups, the difference between the groups was not significant.

There was high muscle mass loss in the first seven days after surgery. The average percentage of muscle mass lost within the weight loss decreased from the 7th postoperative day to the 90th day. It was found that the average percentage of fat mass lost increased. In individuals in BSD + PS, a weight loss of 6.1% was found on the 7th postoperative day and 22.8% at the end of the 90th postoperative day. The percentage of fat mass within the average weight lost up to the 90th day was 68.3% and the percentage of muscle mass was 29.9%. In individuals in BSD, 22.6% of the current weight was lost after the 90th day. In individuals in BSD, the percentage of fat mass lost within the weight lost at the end of the 90th day was 66.7% and the percentage of muscle mass was 31.5%. It was found that women in BSD + PS (40.2%) lost a higher percentage of muscle mass within the weight lost in the first month compared to men (31.7%) (Table 3). No statistically significant difference was found between the groups in terms of lost body weight, muscle mass and fat mass measurements (*p* > 0.05). Time-dependent changes and comparisons of body composition measurements are shown in Figure 1.

**Table 3:** The mean and standard deviation (X ± SD) values of the general and gender-based anthropometric difference measurements of individuals.

		BSD + PS (*n* = 30)	BSD (*n* = 30)	*p*	BSD + PS (*n* = 30)	BSD (*n* = 30)	*p*	BSD + PS (*n* = 30)	BSD (*n* = 30)	*p*
		X̄±SS	X̄ ±SS		X̄±SS	X̄ ±SS		X̄±SS	X̄ ±SS	
		General			Female			Male		
Lost body weight (kg)	Post Op Day 7	6.8 ± 2.13	7.0 ± 3.50	0.766	6.6 ± 1.97	5.6 ± 2.48	0.176	8.5 ± 2.64	9.5 ± 3.78	0.661
	Post Op Day 30	12.8 ± 4.65	14.2 ± 6.87	0.341	12.0 ± 4.41	12.1 ± 3.23	0.940	17.4 ± 3.61	17.8 ± 9.78	0.940
	Post Op Day 90	25.3 ± 6.41	27.2 ± 9.1	0.358	24.5 ± 6.39	23.7 ± 5.33	0.652	30.0 ± 4.78	33.1 ± 11.30	0.473
Body weight lost (%)	Post Op Day 7	6.1 ± 1.64	5.9 ± 2.79	0.714	6.2 ± 1.65	5.2 ± 2.24	0.088	6.0 ± 1.79	7.2 ± 3.24	0.476
	Post Op Day 30	11.3 ± 2.94	11.7 ± 3.72	0.677	11.1 ± 2.86	11.0 ± 3.13	0.914	12.4 ± 3.63	12.8 ± 4.53	0.889
	Post Op Day 90	22.8 ± 4.15	22.6 ± 4.7	0.865	23.1 ± 4.31	21.4 ± 3.89	0.198	21.0 ± 2.55	24.6 ± 5.45	0.235
Fat mass lost (kg)	Post Op Day 7	1.8 ± 2.29	2.0 ± 2.49	0.719	1.6 ± 2.37	1.5 ± 1.87	0.891	3.1 ± 1.07	2.9 ± 3.20	0.902
	Post Op Day 30	7.7 ± 3.8	7.8 ± 3.25	0.910	7.1 ± 3.21	7.2 ± 2.57	0.886	11.9 ± 5.14	8.9 ± 4.10	0.254
	Post Op Day 90	17.2 ± 4.56	17.9 ± 5.3	0.634	16.7 ± 4.46	16.3 ± 3.76	0.711	20.5 ± 4.37	20.6 ± 6.57	0.977
Percentage of fat mass lost in weight lost (%)	Post Op Day 7	31.9 ± 29.3	26.1 ± 25.1	0.564	25.2 ± 39.45	27.5 ± 37.20	0.311	36.6 ± 5.48	29.8 ± 29.25	0.663
	Post Op Day 30	56.7 ± 10.2	55.8 ± 54.1	0.842	58.9 ± 22.42	59.1 ± 14.58	0.971	66.5 ± 14.68	52.9 ± 9.80	0.056
	Post Op Day 90	68.3 ± 8.54	66.7 ± 8.22	0.448	68.4 ± 7.99	68.9 ± 8.65	0.837	68.2 ± 10.98	62.9 ± 7.20	0.290
Muscle mass lost (kg)	Post Op Day 7	4.8 ± 2.66	4.8 ± 3.05	0.993	4.7 ± 2.81	3.9 ± 2.49	0.332	5.1 ± 1.63	6.2 ± 3.47	0.552
	Post Op Day 30	4.6 ± 3.2	5.0 ± 3.8	0.496	4.8 ± 2.66	4.7 ± 2.29	0.833	5.1 ± 1.52	8.4 ± 5.67	0.289
	Post Op Day 90	7.6 ± 2.86	8.8 ± 4.53	0.221	7.4 ± 2.79	7.1 ± 3.07	0.732	9.0 ± 13.28	11.8 ± 5.16	0.260
Percentage of muscle mass lost in weight lost (%)	Post Op Day 7	65.0 ± 5.1	69.6 ± 31.2	0.589	70.7 ± 37.41	72.2 ± 38.54	0.314	60.2 ± 4.67	66.8 ± 28.82	0.664
	Post Op Day 30	39.0 ± 15.98	40.6 ± 12.66	0.673	40.2 ± 16.27	38.5 ± 13.83	0.713	31.7 ± 13.45	44.3 ± 9.83	0.065
	Post Op Day 90	29.9 ± 8.02	31.5 ± 8.07	0.457	29.9 ± 7.86	29.4 ± 8.15	0.850	30.2 ± 10.38	35.0 ± 6.88	0.309

The “Independent Sample-*t*” test (*t*-Chart value) statistics were used to compare two independent groups with normal distribution. The *p* values considered statistically significant are indicated in bold (*p* < 0.05). BMI, Body Mass Index; BSD, Post-bariatric Surgery Diet Group; BSD + PS, Post-bariatric Surgery Diet Group + Protein Supplement Group.

### 3.6 Daily energy and macronutrient intake averages and percentage of DRI coverage

It was found that the averages of energy and macronutrient intake in both groups decreased in the first seven days and increased again over time. In addition, the mean daily energy intake of individuals in BSD + PS was found to be significantly higher than that of BSD on the 90th post-op day (*p* < 0.05). The mean daily protein intake of individuals in BSD + PS was 72.8 ± 31.42 grams (g) in the pre-op period, 25.7 ± 8.53 g on post-op day 7, 33.1 ± 11.95 g on post-op day 30 and 39.5 ± 17.66 g on post-op day 90. Those in BSD were 78.4 ± 34.04 g pre-op, 12.0 ± 12.36 g post-op day 7, 16.2 ± 9.06 g post-op day 30 and 19.5 ± 10.12 g post-op day 90. In addition, the mean protein intake of the BSD + PS group was significantly higher than the BSD group on post-op day 7, 30 and 90 (*p* < 0.05). The mean protein intake of both groups decreased after surgery. The mean intake increased with time but remained below the targeted amount (Table 4).

**Table 4:** Mean daily energy and macronutrient intake of individuals and percentage of DRI coverage.

		Pre-Op	Post Op Day 7	Post Op Day 30	Post Op Day 90
Energy (kcal)	BSD + PS (*n* = 30)	2555.7 ± 806.24	418.2 ± 273.39	439.8 ± 210.71	**549.5 ± 238.06**
	BSD (*n* = 30)	2457.8 ± 913.92	361.7 ± 208.80	384.9 ± 205.93	**392.3 ± 186.67**
Protein (g)	BSD + PS (*n* = 30)	72.8 ± 31.42	**25.7 ± 8.53**	**33.1 ± 11.95**	**39.5 ± 17.66**
	BSD (*n* = 30)	78.4 ± 34.04	**12.0 ± 12.36**	**16.2 ± 9.06**	**19.5 ± 10.12**
Protein (TE %)	BSD + PS (*n* = 30)	11.4 ± 9.00	**24.0 ± 11.44**	**33.5 ± 10.20**	25.9 ± 39.35
	BSD (*n* = 30)	11.9 ± 11.95	**12.5 ± 8.54**	**17.7 ± 5.87**	18.9 ± 1.76
Protein content per weight (g/kg)	BSD + PS (*n* = 30)	0.6 ± 0.70	**0.2 ± 0.11**	**0.3 ± 0.12**	**0.4 ± 0.21**
	BSD (*n* = 30)	0.6 ± 1.10	**0.1 ± 0.11**	**0.2 ± 0.13**	**0.2 ± 0.12**
Carbohydrate (g)	BSD + PS (*n* = 30)	268.6 ± 102.56	34.9 ± 154.50	20.2 ± 48.90	38.2 ± 20.32
	BSD (*n* = 30)	260.6 ± 105.74	40.0 ± 144.45	26.3 ± 102.75	28.5 ± 19.00
Carbohydrate (TE %)	BSD + PS (*n* = 30)	42.0 ± 8.87	**36.7 ± 14.65**	**20.8 ± 9.76**	**28.0 ± 7.65**
	BSD (*n* = 30)	43.2 ± 11.34	**55.7 ± 20.23**	**33.2 ± 15.65**	**32.8 ± 12.66**
Fat (g)	BSD + PS (*n* = 30)	122.7 ± 42.47	12.4 ± 19.05	23.6 ± 13.62	**26.5 ± 14.57**
	BSD (*n* = 30)	118.8 ± 52.39	10.7 ± 29.05	19.9 ± 10.09	**19.6 ± 11.49**
Fat (TE %)	BSD + PS (*n* = 30)	43.4 ± 8.03	30.9 ± 13.23	46.7 ± 13.89	40.8 ± 13.03
	BSD (*n* = 30)	43.0 ± 9.87	30.8 ± 15.32	47.9 ± 13.43	45.7 ± 15.05
Energy (DRI coverage)	BSD + PS (*n* = 30)	103.8 ± 32.1	17.5 ± 11.9	18.1 ± 9.2	**22.7** ± **10.3**
	BSD (*n* = 30)	102.1 ± 38.6	16.0 ± 39.3	16.0 ± 8.7	**17.1** ± **8.0**
Protein (DRI coverage)	BSD + PS (*n* = 30)	150.8 ± 61.5	**53.3** ± **19.0**	**63.5** ± **24.7**	**68.6** ± (30.4–182.6)*
	BSD (*n* = 30)	159.2 ± 65.0	**25.4** ± **27.0**	**33.6** ± **17.1**	**34.6** ± (9.1–103.8)*
Carbohydrate (DRIcoverage)	BSD + PS (*n* = 30)	206.6 ± 78.9	23.7 ± (6.6 + 117.3)*	15.5 ± (4.9 + 70.3)*	29.4 ± 15.6
	BSD (*n* = 30)	200.5 ± 81.3	30.8 ± (5.2 + 207.1)*	20.2 ± (2.9–155.2)*	21.9 ± 14.6
Fat (DRIcoverage)	BSD + PS (*n* = 30)	122.73 ± 42.47	13.93 ± 8.14	30.00 ± 11.03	26.45 ± 14.54
	BSD (*n* = 30)	118.76 ± 52.39	13.20 ± **11.80**	36.87 ± 17.56	19.56 ± 11.49

The “Independent Sample-*t*” test (*t*-Chart) is used to compare two independent groups with a normal distribution. *The “Mann–Whitney U” test (Z Chart value) for comparing two independent groups that do not have a normal distribution. Statistics were used. The *p* values considered statistically significant are indicated in bold (*p* < 0.05). BSD, Post-bariatric Surgery Diet Group; BSD + PS, Post-bariatric Surgery Diet Group + Protein Supplement Group; TE, Total Energy; g, Gram; kg, Kilogram.

### 3.7 Mean daily vitamin and mineral intake of individuals and percentage of DRI coverage

It was observed that the average daily dietary vitamin and mineral intake of the individuals decreased at the end of the 90th day post-op compared to the beginning. However, the average intake increased over time (Table 5). When the difference between the groups was examined, the average potassium mineral, riboflavin, folic acid, vitamin A and C intakes of the individuals in BSD + PS were significantly higher than those in BSD on the 90th day post-op (*p* < 0.05). The percentages of meeting the DRI according to the groups differed depending on time. The percentage of meeting the vitamins of the individuals in BSD + PS was found to be higher than those in BSD. However, it was observed that the vitamin and mineral intake rates of the individuals in both groups could not be met according to the DRI. When the difference between the groups was examined, the average A, C vitamin and calcium, potassium mineral intakes of the individuals in BSD + PS were significantly higher than those in BSD on the 90th day post-op (*p* < 0.05).

**Table 5:** Mean daily vitamin and mineral intake of individuals.

Micronutrients		Pre-Op	Post Op Day 7	Post Op Day 30	Post Op Day 90
Thiamine vit. (B1) (mg)	BSD + PS (*n* = 30)	1.4 ± 0.81	0.2 ± (0.76–0.08)	0.2 ± 0.17	0.3 ± 0.16
	BSD (*n* = 30)	1.2 ± 0.63	0.1 ± (0.00–0.70)	0.2 ± 0.14	0.2 ± 0.11
Riboflavin vit. (B2) (mg)	BSD + PS (*n* = 30)	1.5 ± 0.48	0.4 ± (0.00–1.30)	0.5 ± 0.34	0.6 ± 0.38*
	BSD (*n* = 30)	1.5 ± 0.55	0.4 ± (0.00–1.90)	0.4 ± 025	0.5 ± 0.23*
Pantothenic acid vit. (B5) (mg)	BSD + PS (*n* = 30)	4.8 ± 1.67	1.0 ± 0.67	1.3 ± 0.77	1.3 ± 0.78
	BSD (*n* = 30)	4.5 ± 1.72	1.0 ± 0.64	1.1 ± 0.54	1.2 ± 0.64
Pyridoxine vit. (B6) (mg)	BSD + PS (*n* = 30)	1.4 ± 0.58	0.2 ± (0.00–1.10)	0.3 ± 0.18	0.3 ± (0.10–0.90)
	BSD (*n* = 30)	1.3 ± 0.57	0.2 ± (0.10–0.70)	0.2 ± 0.18	0.4 ± (0.00–1.40)
Biotin vit. (B7) (mcg)	BSD + PS (*n* = 30)	48.8 ± 24.72	10.3 ± 6.94	15.8 ± 9.68	14.8 ± 10.67
	BSD (*n* = 30)	49.4 ± 24.88	12.2 ± 7.92	12.3 ± 7.01	13.4 ± 8.48
Cobalamin vit. (B12) (mcg)	BSD + PS (*n* = 30)	4.4 ± 1.90	0.9 ± (0.00–2.60)	1.4 ± (0.00–4.00)	1.2 ± (0.20–8.90)
	BSD (*n* = 30)	4.4 ± 2.19	0.6 ± (0.00–4.30)	1.0 ± (0.00–9.40)	1.2 ± (0.10–8.80)
Total folic acid vit. (B9) (mcg)	BSD + PS (*n* = 30)	283.6 ± 110.09	32.9 ± (6.60–74.70)	57.2 ± (5.80–273.90)	66.4 ± (24.70–259.30)*
	BSD (*n* = 30)	255.9 ± 110.92	26.6 ± (9.10–161.20)	54.3 ± (14.80–321.90)	61.5 ± (9.60–188.60)*
Vit. C (mg)	BSD + PS (*n* = 30)	79.7 ± 52.35	14.2 ± (0.20–36.60)	21.9 ± (0.30–88.00)	35.3 ± (1.40–134.30)*
	BSD (*n* = 30)	76.3 ± 56.24	13.5 ± (2.00–130.30)	15.3 ± (1.00–62.10)	15.6 ± (0.00–113.30)*
Vit. A (mcg)	BSD + PS (*n* = 30)	1373.5 ± 638.62	195.8 ± (17.70–4219.00)	300.1 ± 159.05	355.0 ± 197.79*
	BSD (*n* = 30)	1286.7 ± 667.24	106.3 ± (18.1–1208.90)	248.7 ± 144.02	266.8 ± 133.13*
Vit. K (mcg)	BSD + PS (*n* = 30)	97.0 ± (24.5–959.1)	16.7 ± (2.40–123.20)	13.1 ± (0.00–213.10)	17.6 ± (0.30–238.80)
	BSD (*n* = 30)	75.4 ± (12.1–959.1)	12.0 ± (0.00–389.80)	13.6 ± (0.90–165.50)	14.5 ± (0.00–65.10)
Iron (mg)	BSD + PS (*n* = 30)	15.1 ± 7.90	1.2 ± (0.20–9.40)	1.9 ± (0.50–6.10)	2.9 ± 1.87
	BSD (*n* = 30)	14.5 ± 7.32	1.3 ± (0.20–5.60)	2.0 ± (0.60–8.90)	2.3 ± 1.54
Calcium (mg)	BSD + PS (*n* = 30)	736.9 ± 274.18	167 ± (13.9–869.5)	252.0 ± 222.32	227.8 ± (30.9–1193.7)
	BSD (*n* = 30)	688.1 ± 319.28	174.3 ± (21.00–1450.0)	136.5 ± 119.92	207.4 ± (26.90–420.40)
Zinc (mg)	BSD + PS (*n* = 30)	13.3 ± 5.42	1.3 ± (0.10–4.90)	2.8 ± 1.77	2.9 ± (0.40–15.60)
	BSD (*n* = 30)	13.3 ± 6.26	1.5 ± (0.20–12.80)	2.8 ± 1.96	2.9 ± (0.40–15.50)
Sodium (mg)	BSD + PS (*n* = 30)	4501.0 ± 1682.49	531.1 ± 26.58	591.9 ± 30.07	681.1 ± 30.93
	BSD (*n* = 30)	4353.7 ± 2196.61	297.8 ± 34.42	458.7 ± 27.65	628.4 ± 33.35
Potassium (mg)	BSD + PS (*n* = 30)	2544.2 ± 1095.72	450.5 ± (80.8–2087.0)	535.2 ± 374.60	642.4 ± (198.9–2117.8)*
	BSD (*n* = 30)	2433.7 ± 1045.01	502.0 ± (24.20–2208.0)	419.9 ± 271.71	589.1 ± (87.00–957.70)*
Magnesium (mg)	BSD + PS (*n* = 30)	383.7 ± 212.79	51.5 ± 37.43	59.4 ± 45.8	73.0 ± 45.8
	BSD (*n* = 30)	358.6 ± 203.40	53.0 ± 43.12	55.6 ± 26.7	59.0 ± 32.9
Micronutrients Percentage of DRI coverage		*Pre-Op*	*Post Op Day 7*	*Post Op Day 30*	*Post Op Day 90*
Thiamine vit. (B1) (mg)	BSD + PS (*n* = 30)	122.4 ± 69.96	12.8 ± (0.00–45.45)	19.8 ± 14.72	23.4 ± 14.56
	BSD (*n* = 30)	105.6 ± 53.71	9.0 ± (0.00–63.64)	17.2 ± 12.19	19.3 ± 10.09
Riboflavin vit. (B2) (mg)	BSD + PS (*n* = 30)	128.5 ± 41.14	34.8 ± (0.00–118.18)	44.2 ± 30.68	50.6 ± 33.70*
	BSD (*n* = 30)	129.2 ± 49.44	31.8 ± (0.00–172.73)	35.4 ± 21.89	40.5 ± 20.01*
Pantothenic acid vit. (B5) (mg)	BSD + PS (*n* = 30)	4.8 ± 1.67	1.0 ± 0.67	1.3 ± 0.77	1.3 ± 0.78
	BSD (*n* = 30)	4.5 ± 1.72	1.0 ± 0.64	1.1 ± 0.54	1.2 ± 0.64
Pyridoxine vit. (B6) (mg)	BSD + PS (*n* = 30)	108.2 ± 44.88	15.3 ± (0.00 ± 84.62)	21.4 ± 13.80	23.0 ± (7.69–69.23)
	BSD (*n* = 30)	100.1 ± 44.07	15.3 ± (7.69 ± 53.85)	15.4 ± 13.89	26.3 ± (0.00–107.69)
Biotin vit. (B7) (mcg)	BSD + PS (*n* = 30)	162.7 ± 82.40	34.2 ± 23.12	52.5 ± 32.26	49.2 ± 35.58
	BSD (*n* = 30)	164.6 ± 82.93	40.6 ± 26.41	41.1 ± 23.35	44.3 ± 28.25
Cobalamin vit. (B12) (mcg)	BSD + PS (*n* = 30)	184.8 ± 79.03	37.5 ± (0.00–108.33)	56.2 ± (0.00–166.67)	50.0 ± (8.33–370.83)
	BSD (*n* = 30)	182.7 ± 91.36	25.0 ± (0.00–179.17)	41.0 ± (0.00–391.67)	50.6 ± (4.17–366.67)
Total folic acid vit. (B9) (mcg)	BSD + PS (*n* = 30)	70.8 ± 27.52	8.2 ± (1.65–18.68)	14.3 ± (1.45–68.48)	16.5 ± (6.18–64.83)*
	BSD (*n* = 30)	63.9 ± 27.77	6.6 ± (2.28–40.30)	13.3 ± (3.70–80.48)	15.5 ± (2.40–47.15)*
Vit. C (mg)	BSD + PS (*n* = 30)	100.1 ± 65.79	17.8 ± (0.27–48.80)	21.0 ± (0.33–117.33)	**46.6 ± (1.87–179.07)**
	BSD (*n* = 30)	99.2 ± 75.73	16.1 ± (2.22–173.73)	20.8 ± (1.33–82.80)	**18.0 ± (0.00–151.07)**
Vit. A (mcg)	BSD + PS (*n* = 30)	1373.5 ± 638.62	195.8 ± (17.70–4219.00)	300.1 ± 159.05	355.0 ± 197.79*
	BSD (*n* = 30)	1286.7 ± 667.24	106.3 ± (18.1–1208.90)	248.7 ± 144.02	266.8 ± 133.13*
Vit. K (mcg)	BSD + PS (*n* = 30)	99.5 ± (20.42–799.58)	17.2 ± (2.67–136.89)	12.2 ± (0.00–225.67)	19.2 ± (0.33–265.33)
	BSD (*n* = 30)	75.7 ± (13.44–1065.67)	12.3 ± (0.00–433.11)	13.8 ± (1.00–183.89)	13.5 ± (0.00–72.33)
Iron (mg)	BSD + PS (*n* = 30)	123.4 ± 87.07	10.0 ± (1.11–52.22)	17.3 ± 12.46	18.3 ± (6.67–85.00)
	BSD (*n* = 30)	109.7 ± 76.06	8.9 ± (1.11–31.11)	13.9 ± 15.61	18.9 ± (3.89–81.25)
Calcium (mg)	BSD + PS (*n* = 30)	72.9 ± 27.40	16.7 ± (1.39–86.95)	25.0 ± 22.27	22.8 ± (3.09–119.37)*
	BSD (*n* = 30)	68.8 ± 32.01	17.4 ± (2.10–145.00)	13.7 ± 12.04	20.0 ± (2.69–42.04)*
Zinc (mg)	BSD + PS (*n* = 30)	149.2 ± 56.4	14.3 ± (1.25–61.25)	32.2 ± 22.19	34.9 ± (5.00–182.50)
	BSD (*n* = 30)	153.7 ± 67.42	16.9 ± (2.50–160.00)	31.5 ± 21.91	33.6 ± (5.00–140.91)
Sodium (mg)	BSD + PS (*n* = 30)	300.1 ± 112.17	35.4 ± 26.58	39.4 ± 30.07	45.5 ± 27.65
	BSD (*n* = 30)	290.3 ± 146.44	19.9 ± 34.42	30.9 ± 30.93	41.6 ± 33.35
Potassium (mg)	BSD + PS (*n* = 30)	90.1 ± 38.03	17.3 ± (2.57–80.27)	19.2 ± 13.82	23.2 ± (7.52–81.45)*
	BSD (*n* = 30)	88.8 ± 38.72	19.3 ± (0.93–84.92)	14.3 ± 9.09	21.05 ± (3.35–35.60)*
Magnesium (mg)	BSD + PS (*n* = 30)	110.5 ± 57.31	15.5 ± 11.96	17.5 ± 13.89	22.0 ± 15.13
	BSD (*n* = 30)	106.1 ± 54.42	16.2 ± 13.63	16.9 ± 7.99	18.8 ± 10.56

The “Independent Sample-*t*” test (*t*-Chart) is used to compare two independent groups with a normal distribution. *The “Mann–Whitney U” test (Z Chart value) for comparing two independent groups that do not have a normal distribution. Statistics were used. The *p* values considered statistically significant are indicated in bold (*p* < 0.05). BSD, Post-bariatric Surgery Diet Group; BSD + PS, Post-bariatric Surgery Diet Group + Protein Supplement Group; vit, Vitamin; mg, Milligram; mcg, Microgram.

## 4 Discussion

Our study, which aimed to evaluate the success of protein supplements recommended in addition to postoperative nutrition education and their effect on muscle mass, was conducted with 60 individuals aged 19–60 years who underwent sleeve gastrectomy (SG) surgery and had similar characteristics. The groups were divided into two groups as protein supplement users (BSD + PS) and non-users (BSD). There was no significant difference between the groups in terms of muscle mass in the postoperative period. Although supplementation increased protein intake, we found that it caused compliance difficulties due to different reasons. In addition, we observed that not all individuals were able to complete the targeted daily intake. We determined that more comprehensive, strictly monitored and nutrition education prepared by expert dietitians is required before protein supplementation is considered.

High weight loss in postoperative processes may lead to undesirable loss of muscle mass (43). Since it has a great effect on the regulation of metabolic balance, preservation of skeletal muscle integrity and functional capacity according to age, precautions should be taken (21). Surgery can realize the expected weight loss with new arrangements in the anatomical structure (44). However, the intake, digestion and absorption of many nutrients, especially amino acids, change due to these new arrangements and cause nutritional deficiencies. One of the major reasons for this has been defined as a decrease in gastric volume and inadequate release of digestive enzymes, especially hydrochloric acid (15). It has also been observed that taste and odor sensitivity may develop in the postoperative period, especially in the first month (13).

In a study by Andreu et al. examining the effect of protein intake on muscle mass and blood parameters, it was found that supplement use despite recommendations was 63.4, 50.5, and 33.7% at 4th, 8th, and 12th months, respectively. In addition, 45, 35, and 37% of the targeted protein intake was <60 g/day at 4th, 8th, and 12th months, respectively. Male gender and weight loss were significantly associated with muscle mass independent of protein intake. It was emphasized that compliance with targeted protein supplements was poor (14). Another study supporting this situation was revealed in a study conducted by Bertoni et al. (45). Assessed protein intake in the first three months after sleeve gastrectomy (SG) in 47 patients with severe obesity. It found that protein intake from foods was insufficient, averaging 30.0 g/day in the first month and increasing to 34.9 g/day (*p* = 0.003) by the third month, both below the recommended 60 g/day. The use of protein supplementation significantly increased total protein intake to 42.3 g/day (*p* < 0.001) in the first month and 39.6 g/day (*p* = 0.002) in the third month, but compliance with supplementation was low, dropping from 63.8 to 21.3%. Overall, the study concluded that despite dietary guidance and supplementation, protein intake remained inadequate in the early post-operative period (45). In our study, there was a significant increase in the protein intake of both groups at the end of 3 months, but the recommended protein intake was not reached. Literature data support our study.

In a 2016 study by Schollenberger et al. (39) researchers examined the effect of 15 g/day protein supplementation in addition to standard nutritional therapy on body weight, fat mass loss, and muscle mass preservation after surgery. The results showed that protein intake amounts were significantly higher in the protein supplementation group compared to the control group (*p* < 0.001). At the 6-month follow-up, fat mass loss was found to be significantly greater in the protein supplementation group (79%) compared to the control group (73%) (*p* = 0.02). Additionally, muscle mass loss was less significant in the protein supplementation group (21%) compared to the control group (27%) (*p* = 0.05). However, when analyzing overall body weight loss, the results were similar between the protein supplementation group (25–7.2%) and the control group (20.9–3.9%) (*p* > 0.05).

In a study examining the effects of high protein (2 g/kg/day) and standard protein (1 g/kg/day) diets on anthropometric measurements after surgery, the high protein group showed a significant decrease in fat mass from the 3rd month (*p* < 0.01), while muscle mass and basal metabolic rate were preserved (*p* < 0.01). Another study also found that protein supplementation prevented muscle loss. In the group receiving 1.2 g/kg/day protein supplementation in the 1st month, muscle mass at the end of the 6^th^ month was similar to the control group (46). In our study, we also found a significant decrease in muscle loss over time with protein supplementation, but no difference between the groups (Group: *F* = 3.297; *p* = 0.075, time: *F* = 202.784; *p* < 0.001, group*time; *F* = 0.317; *p* = 0.743).

Supplements may be considered to have an indirect effect by helping to support reaching the targeted intake, but the expected adequate protein intake (<60 g/day) could not be reached in nutritional therapy due to reasons such as the 3-month follow-up of the study due to pandemic conditions, the possibility of food intolerance in individuals, gastrointestinal complaints caused by some foods, shrinking stomach volume and changing hormonal systems.

In the study conducted by Dagan et al. (47) in 2016, when the average energy intake of all individuals followed up was examined, it was found to be 2117.6 ± 920.9 kcal in the preoperative period, 838.9 ± 348.5 kcal in the 3rd month, 1105.9 ± 453.7 kcal in the 6th month and 1296.5 ± 496.5 kcal in the 12th month. When the daily carbohydrate intake values were examined, it was found to be 222.2 ± 125.8 g in the preoperative period, 90.8 ± 47 g in the 3rd month, 121.1 ± 67.2 g in the 6th month and 142.3 ± 69.8 g in the 12th month. When the average daily fat intake was examined, it was found to be 90.4 ± 43.9 g in the preoperative period, 32.7 ± 16.9 g in the 3rd month, 43.9 ± 19.4 g in the 6th month and 52.4 ± 22.6 g in the 12th month. When the daily protein intake was examined, it was found to be 93.5 g in the preoperative period, 41.4 ± 18.5 g in the 3rd month, 51.7 ± 18.9 g in the 6th month and 58.1 ± 22.9 g in the 12th month.

In a study conducted by Giusti et al. (48) in 2015, the average daily energy intake of 16 women decreased from 2072 ± 108 kcal preoperatively to 681 ± 58 kcal in the 1st month, then gradually increased to 1.448 ± 57 kcal by the 36th month. Average daily protein intake declined from 87 ± 4 g preoperatively to 29 ± 2 g in the 1st month, later reaching 57 ± 3 g at 36 months. Carbohydrate intake dropped from 231 ± 11 g preoperatively to 76 ± 8 g in the 1st month, then increased to 144 ± 6 g by 36 months. Similarly, fat intake decreased from 89.7 ± 7 g preoperatively to 29 ± 3 g in the 1st month, before rising to 76 ± 5 g at 36 months. Overall, significant decreases in energy, protein, carbohydrate and fat intake were observed in the early postoperative period, with gradual increases over the long-term.

In the study conducted by Gobato et al.’s (49) in 2014, study found that pre-operative daily energy intake was 1812.2 ± 767.52 kcal, decreasing to 1610.3 ± 322.25 kcal by the 6th month. Daily protein intake also decreased from 96.2 ± 36.13 g pre-operatively to 47.1 ± 17.70 g at 6 months. The average daily energy intake was higher at 2555.7 ± 806.24 kcal in the BSD + PS group and 2457.8 ± 913.92 kcal in the BSD group, compared to other studies. Daily zinc intake decreased from 10.0 ± 4.01 mg pre-operatively to 6.9 ± 3.25 mg at 6 months. Intakes of vitamins and minerals like vitamin B12, iron, calcium, magnesium, and folic acid also decreased significantly from pre-operative to 6-month levels. In our study, it was determined that the iron, calcium, zinc, magnesium and potassium mineral intake averages were below the targeted values in all periods, and similarly, vitamins, especially vitamin A, vitamin C, thiamine and riboflavin, fell below the targeted values in the 1st and 3rd months after surgery.

The existing literature supports that protein intake is inadequate in the postoperative period and adherence to protein supplementation is low. The most important limitations of our study are the small sample size and the relatively short follow-up period. We focused our attention on the first period after surgery, when the protein intake issue is more important and weight loss is faster. In order to better evaluate the clinical impact of our results, they can be supported by further studies with longer follow-up and body composition data. However, our sample was mostly limited to female gender and adult patients, and the physical activity levels recorded in the first three months after surgery were very low. In our study, we evaluated only patients treated with SG, and we can assume that postoperative protein intake may vary in different types of bariatric surgery.

## 5 Conclusion

In post-bariatric surgery patients, muscle mass preservation is highly related to protein intake. However, in our prospective study, the targeted daily minimum protein intake level could not be reached even with protein supplements. No effect of daily protein intake with protein supplements on the decrease in muscle mass loss or increase in fat mass loss was detected during the 3-month follow-up period. In addition, undesirable reasons such as the fact that protein supplements consumed by individuals may cause gastrointestinal complaints, the feeling of boredom caused by daily use of these flavors, and the inability to maintain consumption discipline made it difficult to comply with the targeted protein intake recommendations. For this reason, it was observed that the targeted daily intake amounts of other food groups could not be reached. In addition to the protein malnutrition experienced by post-bariatric surgery patients, vitamin and mineral losses were found to be significantly low. This study also raises awareness among existing healthcare providers who need to encourage adequate protein intake in post-bariatric surgery patients. It was concluded that new postoperative diet models that include more rigorous and intensive training programs that take into account all food groups, especially protein, are needed before considering supplements to minimize muscle mass losses. Studies investigating the quantity (g/day) and quality (whey, casein or soy) of protein supplements or high-protein diets in these models in larger study populations are needed.

## Data Availability

The raw data supporting the conclusions of this article will be made available by the authors, without undue reservation.
